# Aerobactin Seems To Be a Promising Marker Compared With Unstable RmpA2 for the Identification of Hypervirulent Carbapenem-Resistant *Klebsiella pneumoniae*: *In Silico* and *In Vitro* Evidence

**DOI:** 10.3389/fcimb.2021.709681

**Published:** 2021-09-13

**Authors:** Chaitra Shankar, Soumya Basu, Binesh Lal, Sathiya Shanmugam, Karthick Vasudevan, Purva Mathur, Sudha Ramaiah, Anand Anbarasu, Balaji Veeraraghavan

**Affiliations:** ^1^Department of Clinical Microbiology, Christian Medical College and Hospital, Vellore, India; ^2^Medical & Biological Computing Laboratory, School of Biosciences & Technology, Vellore Institute of Technology, Vellore, India; ^3^Department of Laboratory Medicine, Jai Prakash Narayan Apex, Trauma Centre, All India Institute of Medical Sciences, New Delhi, India

**Keywords:** *Klebsiella pneumoniae*, hypervirulent, aerobactin, *rmpA2*, carbapenem resistance, OXA-232, structure, stability

## Abstract

**Background:**

The incidence of hypervirulent (hv) carbapenem-resistant (CR) *Klebsiella pneumoniae* (Kp) is increasing globally among various clones and is also responsible for nosocomial infections. The CR-hvKp is formed by the uptake of a virulence plasmid by endemic high-risk clones or by the uptake of plasmids carrying antimicrobial resistance genes by the virulent clones. Here, we describe CR-hvKp from India belonging to high-risk clones that have acquired a virulence plasmid and are phenotypically unidentified due to lack of hypermucoviscosity.

**Methods:**

Twenty-seven CRKp isolates were identified to possess *rmpA2* by whole-genome sequencing; and resistance and virulence determinants were characterized. By *in silico* protein modeling (and validation), protein backbone stability analysis, and coarse dynamics study, the fitness of RmpA, RmpA2, and aerobactin-associated proteins-IucA and IutA, were determined to establish a reliable marker for clinical identification of CR-hvKp.

**Results:**

The CR-hvKp belonged to multidrug-resistant (MDR) high-risk clones such as CG11, CG43, ST15, and ST231 and carried OXA-232 as the predominant carbapenemase followed by NDM. The virulence plasmid belonged to IncHI1B replicon type and carried frameshifted and truncated *rmpA* and *rmpA2*. This resulted in a lack of hypermucoviscous phenotype. However, functional aerobactin was expressed in all high-risk clones. *In silico* analysis portrayed that IucA and IutA were more stable than classical RmpA. Furthermore, IucA and IutA had lower conformational fluctuations in the functional domains than the non-functional RmpA2, which increases the fitness cost of the latter for its maintenance and expression among CR-hvKp. Hence, RmpA and RmpA2 are likely to be lost among CR-hvKp owing to the increased fitness cost while coding for essential antimicrobial resistance and virulence factors.

**Conclusion:**

Increasing incidence of convergence of AMR and virulence is observed among *K. pneumoniae* globally, which warrants the need for reliable markers for identifying CR-hvKp. The presence of non-functional RmpA2 among high-risk clones highlights the significance of molecular identification of CR-hvKp. The negative string test due to non-functional RmpA2 among CR-hvKp isolates challenges phenotypic screening and faster identification of this pathotype. This can potentially be counteracted by projecting aerobactin as a stable, constitutively expressed, and functional marker for rapidly evolving CR-hvKp.

## Introduction

Hypervirulent (Hv) *Klebsiella pneumoniae* (Kp) is a notorious pathogen causing a wide spectrum of infections in immunocompetent patients as well as immunosuppressed patients (Shon et al., 2013). This pathotype is being increasingly reported in the last decade among community- and healthcare-associated infections and has acquired antimicrobial resistance, unlike its former counterpart (Zhan et al., 2017; Hao et al., 2020; Li et al., 2021). The virulence plasmid described earlier such as the pLVPK and pK2044 lacked genes coding for antimicrobial resistance and typically belonged to IncHI1B (Chen et al., 2004). However, recent reports suggest the acquisition of virulence plasmids by multidrug-resistant (MDR) Kp and hence insertion of antimicrobial resistance genes (ARGs) such as *bla*_KPC-2_ and *catA1* into the virulence plasmid (Dong et al., 2018; Chen et al., 2020; Shankar et al., 2020a). In addition, reports of fusion/mosaic plasmids that carry two replicons such as IncHI1B–IncFIB and IncFIIK–IncFIBK are emerging, which code for antimicrobial resistance determinants as well as virulence genes (Lam et al., 2019; Turton et al., 2019). Furthermore, HvKp is no longer confined to selected clones but is reported among regional endemic clones such as ST11 in China and ST147 in Europe (Turton et al., 2019; Li et al., 2021). Hence, the threat of these superbugs is increasing, and this pathotype is evolving at a tremendous pace.

Traditionally, string test, a phenotypic screening marker, was used to identify hvKp, but recent studies have revealed poor sensitivity and specificity in identifying hvKp. Therefore, string test is no longer valuable in the identification of hvKp (Tan et al., 2014). Amidst the various genomic modifications that the hvKp has undergone over the last decade, the markers for genomic identification have remained a constant despite a lack of clear definition. A combination of genes *rmpA*, *rmpA2*, *iucA*, *iroB*, and *iroN* (Russo and MacDonald, 2020; Hu et al., 2021; Russo et al., 2021) has been used in identifying hvKp along with determining the pathogenicity in virulence models using mice or *Galleria mellonella*.

In India, high rates of MDR and extensively drug-resistant (XDR) Kp are frequent causes of nosocomial infections, and they often carry NDM and OXA48-like carbapenemases alone or in combination (Veeraraghavan et al., 2017; Shankar et al., 2021). The common clones of carbapenem-resistant Kp (CRKp) in India include ST231, the endemic clone, and other international high-risk clones such as ST11, ST14, ST15, and ST147 (Shankar et al., 2021). Conventionally, these CRKp do not carry the virulence plasmid; instead, they are characterized by the presence of four to five plasmids that carry ARGs. With advancement of genomics in bacteriology, the convergence of CRKp and HvKp to form CR-HvKp has been identified in various countries including India (Lam et al., 2019; Turton et al., 2019; Tang et al., 2020). In the present study, we report CRKp belonging to high-risk international clones in India that acquired the virulence plasmid-carrying frameshifted *rmpA2*.

Although previous studies have reported the correlation of *rmpA* and *rmpA2* as markers for hypervirulent and hypermucoviscous pathotypes, there is a dearth of comprehensive correlations between the constitutive virulence markers for hvKp, viz., *rmpA*, *rmpA2*, and *aerobactin*, based on “genome-structure” perspectives. Our research group has extensively worked on genomics (Jacob et al., 2019; Shankar et al., 2020a; Vasudevan et al., 2020; Shankar et al., 2021) and *in silico* structural analysis (Lavanya et al., 2013; Lavanya et al., 2014; Lavanya et al., 2015) to decipher the functional circuitry of various pathogenic proteins. The present study adopted a combination of whole-genome sequencing (WGS), genomic analysis, computational modeling, and structural dynamics studies, which have not been used previously to assess the concerned virulence markers as per our knowledge. We aimed to understand the impact of *rmpA2* mutations on the fitness among CR-hvKp based on the comparative structure-function profiles of RmpA, RmpA2, and aerobactin (IucA and IutA). This study will potentially lead to identifying a sustainable molecular marker for CR-hvKp in the context of deleterious mutations and molecular fitness, which can subsequently be used for clinical identification of CR-hvKp strains.

## Materials and Methods

### *In-Vitro* Methods

#### Bacterial Isolates and Clinical Details of Patients

Twenty-seven *K. pneumoniae* identified during 2017–2019 at the Department of Clinical Microbiology, Christian Medical College and Hospital, Vellore, and the Department of Laboratory Medicine, Jai Prakash Narayan Apex, Trauma Centre, AIIMS, New Delhi, were included in the study. The isolates were obtained from clinical specimens such as blood (n = 13), cerebrospinal fluid (n = 4), broncho-alveolar lavage (n = 8), and pus (n = 2). String test was performed to screen for the hypermucoviscous phenotype followed by screening of *rmpA* and *rmpA2* by PCR to determine the hypervirulent strains (Brisse et al., 2009). Antimicrobial susceptibility testing against various first and second-line antimicrobials such as piperacillin/tazobactam, ceftazidime, cefepime, meropenem, imipenem, gentamicin, amikacin, ciprofloxacin, and minocycline was performed by Kirby Bauer disc diffusion and interpreted according to Clinical and Laboratory Standards Institute (CLSI) guidelines (CLSI 2017–2019). The isolates were defined as MDR if they were resistant to more than one agent in at least three classes of antimicrobials (Magiorakos et al., 2012). Colistin susceptibility was determined using broth microdilution to obtain the minimum inhibitory concentrations (MICs) and interpreted according to European Committee on Antimicrobial Susceptibility Testing (EUCAST) guidelines. *In vitro* fitness of the study isolates was assessed using growth curve assay as previously described (Bachman et al., 2015).

Clinical details of the patients were obtained from electronic medical records prospectively, and hence, informed consent could not be obtained. The patient outcomes, therapeutic regimen, duration of hospital stay, comorbidities, and nature of infection were analyzed. Hospital-acquired infection (HAI) was defined as a positive culture with *K. pneumoniae* after 48 h of admission to hospital. Community-acquired infection (CAI) was defined as a positive culture with *K. pneumoniae* before 48 h on admission to hospital (Shankar et al., 2018).

#### DNA Extraction and Genome Sequencing

Total genomic DNA was extracted from the pelleted cells using Wizard DNA purification kit (Promega, WI, USA) as per the manufacturer’s protocol. Extracted DNA was quantified using NanoDrop One spectrophotometry (Thermo Fisher Scientific, MA, USA) and Qubit 3.0 fluorometry (Life Technologies, CA, USA) and stored at −20°C until further use.

Sequencing library was prepared using the Nextra DNA Flex library preparation kit (Illumina, San Diego, CA) as per the manufacturer’s instructions. Subsequently, the paired-end library was subjected to sequencing on a HiSeq 2500 platform (Illumina, USA) generating 2 × 150-bp reads. Sequencing reads with a PHRED quality score below 20 were discarded; and adapters were trimmed using cutadapt v1.8.1 and assessed with FastQC v0.11.4.

Long-read sequencing was carried out using Oxford NanoporeMinION platform with FLO-MIN106 R9 flow cell (Oxford Nanopore Technologies, Oxford, UK). Long-read DNA library was prepared using the SQK-LSK108 ligation sequencing kit (v.R9) along with ONT EXP-NBD103 Native Barcode Expansion kit following the manufacturer’s protocol (Oxford Nanopore Technologies, Oxford, UK). The library was loaded onto the flow cells and run for 48 h using the standard MinKNOW software. The Fast5 files generated from MinION sequencing were subjected to base calling using Guppy (https://github.com/gnetsanet/ONT-GUPPY; accessed in January 2020).

#### Genome Assembly and Evaluation

Draft genome sequence data generated using Illumina were assembled using SPAdes (v.3.13.0) (Bankevich et al., 2012). Complete and highly accurate assembly was achieved using hybrid *de novo* assembly approach (Vasudevan et al., 2020). The nanopore long reads were error-corrected with the standalone Canu error correction tool (v.1.7) and assembled using the Unicycler hybrid assembly pipeline (v 0.4.6) with the default settings (Koren et al., 2017; Wick et al., 2017). The obtained genome sequence was polished using high-quality Illumina reads as described previously (Walker et al., 2014). The assembled complete genome was subjected to quality assessment using CheckM v1.0.5 (Parks et al., 2015) and Quast v4.5 (Gurevich et al., 2013). CheckM estimated the completeness and contiguity, while Quast was used to detect mis-assemblies, mismatches, and indels.

#### Genome Analysis

Genome assemblies were submitted to National Center for Biotechnology Information (NCBI) GenBank and annotated using the Prokaryotic Genome Annotation Pipeline (PGAP v.4.1) from NCBI (Tatusova et al., 2016). The resistance profile of the assembled genome sequences was obtained from Resfinder 4.1 available from CGE server (https://cge.cbs.dtu.dk/services/ResFinder/). Similarly, the presence of plasmids in the genomes was identified and characterized using PlasmidFinder (v.1.3) available at CGE server (https://cge.cbs.dtu.dk/services/PlasmidFinder). Furthermore, MLST and virulence locus (yersiniabactin, aerobactin, and other siderophore production systems) were identified using Kleborate (v.2.0.0) (https://github.com/katholt/Kleborate) (Lam et al., 2021). The presence of virulence factors was confirmed using virulence database at Pasteur Institute for Kp (https://bigsdb.pasteur.fr/cgi-bin/bigsdb/bigsdb.pl?db=pubmlst_klebsiella_seqdef&page=sequenceQuery). The final assembled circular chromosome and plasmid were visualized using CGview server v.1.0 (Grant and Stothard, 2008).

#### Screening of Mutations in *rmpA* and *rmpA2*

The sequences of *rmpA* and *rmpA2* obtained from whole genome were compared with references AUB50662.1 and AAR07704.1, respectively, to determine the mutations. The sequences were aligned using Clustal Omega (https://www.ebi.ac.uk/Tools/msa/clustalo/); and mismatches in nucleotides were identified. The allele numbers of *rmpA* and *rmpA2* were assigned using the database at BIGSdb [Bacterial Isolate Genome Sequence Database] (https://bigsdb.pasteur.fr/cgi-bin/bigsdb/bigsdb.pl?db=pubmlst_klebsiella_seqdef&page=sequenceQuery).

### *In-Silico* Methods

#### Modeling of the Proteins

The genomic study was subsequently correlated with structural profiles of RmpA, RmpA2, IutA, and IucA to obtain an insight into their structural stabilities. However, no crystal structures of RmpA, RmpA2, and IutA were available at present as observed from NCBI-BLASTp search in Protein Data Bank. Hence, intensive computational modeling and validations were employed to predict the structure of the proteins. The sequences of RmpA, RmpA2, IucA, and IutA as well as the mutant varieties of RmpA2 were obtained from our WGS data. In the absence of suitable templates, the entire structures of RmpA, RmpA2, and IutA were modeled using an extended dual-step method. In the first step, the 3D structure prediction (with available sequences) based on homology and threading method was primarily performed by using I-TASSER (https://zhanglab.ccmb.med.umich.edu/I-TASSER/) and RaptorX (http://raptorx.uchicago.edu/) servers using their default parameters. From the server results, the top-ranked models were chosen based on C-scores, B-factors, and low root-mean-square deviation (RMSD) values. C-score (−5 to 2) predicts the global structure accuracy when original structures are unavailable. The modeled proteins that possessed residue-level normalized B-factor trajectories around 0.00 for all structural patterns (helices, strands, and coils) were chosen (Yang and Zhang, 2015; Wang et al., 2016). In the second step of modeling, the server-based models were used as templates to model the final structures of RmpA, RmpA2, and IutA proteins using standalone python-based software MODELLER 10.0. The structures built with MODELLER satisfy spatial restraints expressed as probability density functions and merged with an optimized combination of conjugate gradients and molecular dynamics. This model-building procedure is identical to NMR spectroscopy-based structure elucidation (Webb and Sali, 2017). This extended method of computational modeling comprising homology, threading, and dynamics complied with vital structural parameters and hence minimized local structural errors to improve the quality of predicted model during the unavailability of structural templates (Basu et al., 2021).

Finally, for each of the steepest descent and conjugate gradient parameters, 2,000 steps were performed in Swiss PDB viewer (SPDBV) using GROMOS96 force field *in vacuo* to optimize the modeled structures (Kaplan and Littlejohn, 2001). The structure of IucA was retrieved from Protein Data Bank (ID: 5JM8), and missing residues were added using SPDBV. The sequence of IucA was further aligned with the IucA sequence obtained from WGS data and hence validated. The structures and graphs generated were retrieved from respective sites. The protein structures were visualized in UCSF-CHIMERA (Pettersen et al., 2004).

#### Protein–Structure Validations

The modeled RmpA, RmpA2, and IutA proteins were refined through the GalaxyRefine server (http://galaxy.seoklab.org/index.html) to assess and improve the clash scores, poor rotamers, percentage Ramachandran outliers, and percentage bad side-chain rotamers (Heo et al., 2013). The final models were validated using HARMONY (for RmpA and RmpA2) (http://caps.ncbs.res.in/harmony/) and ProSA-web (for all three modeled proteins) (https://prosa.services.came.sbg.ac.at/prosa.php) servers. HARMONY measures the substitution scores on an individual residue level to assess errors in the protein’s 3D conformation based on folding patterns of previously characterized structures. The substitution graph from HARMONY provides the smoothened scores between query sequences in comparison with the reverse sequences of the respective proteins. The reverse sequence and its scores are used as a control to identify local errors in the proposed protein model. The query sequence should have a higher substitution score than the reverse sequence to avoid possible local errors (Pugalenthi et al., 2006). ProSA-web highlighted protein structure errors based on energy plots and Z-score derived from conformational variations concerning experimentally derived structural patterns (Wiederstein and Sippl, 2007).

#### Protein Backbone Stability Analysis

The backbone stability of the proteins was performed using server DynaMine (http://dynamine.ibsquare.be/). DynaMine predicts backbone flexibility at the residue level in the form of backbone N-H S^2^-order parameter values, which were directly determined from experimental NMR chemical shifts. This S^2^ values portray the restrictions atomic bond movements with respect to the molecular reference frame. A value of 1 means stable conformation (high rigidity), while a value of 0 means fully random bond vector movement (highly dynamic). Furthermore, values >0.8 are referred to as considerably rigid, between 0.6 and 0.7 may depict functionally contextual, and <0.6 denotes highly flexible backbone. DynaMine uses a simple linear regression method to accurately distinguish between folded domains and disordered regions of different magnitudes (Cilia et al., 2014). The average S^2^ scores of the whole proteins and their respective functional domains were determined. The functional domains of the proteins were validated through INTERPRO and Pfam servers.

#### Residue-Level Propensity Analysis Through Coarse Dynamics

CABSflex server (http://biocomp.chem.uw.edu.pl/CABSflex2/) was employed for coarse dynamics study to depict the residue-level fluctuations when compared with the most favorable conformation of the protein. The stabilities were analyzed from the root-mean-square fluctuation (RMSF) values generated based on default restraint parameters. The restraints provided maximum and minimum ranges to pair atoms and contain them within defined spaces with their dynamic orientations. The deviations beyond assigned ranges were designated as unstable. The default settings and restraints were optimized to merge coarse dynamics simulations and consensus protein fluctuations in aqueous environment derived by all-atom molecular dynamics simulation (10-ns timescale with suitable force fields). The default parameters were selected with gap = 3 (minimum distance between previous and next amino acid in the chain to be restrained); minimum and maximum conformational distances were 3.8 and 8.0 Å, respectively (Jamroz et al., 2013).

## Results

### Phenotypic Characterization of *Klebsiella pneumoniae* and Clinical Details of the Patients

All the 27 MDR Kp included in the study were resistant to ceftazidime, cefepime, meropenem, imipenem, amikacin, ciprofloxacin, and minocycline as determined by disc diffusion. Since the isolates were resistant to meropenem and imipenem, they are referred to as carbapenem-resistant (CR-hvKp). Eleven isolates (41%) retained intermediate susceptibility to colistin with MIC of ≤2 µg/ml. Twenty Kp isolates in the study were string test negative, lacking hypermucoviscosity, due to frameshift mutations in *rmpA* and *rmpA2* as mentioned in **Table 1**.

**Table 1:** Results of the whole-genome analysis showing characteristics of carbapenem resistant hypervirulent Kp.

Accession number	*rmpA* and/or *rmpA2*	String test	Capsule type	O antigen	ST	*ybt*, ICEKp	Resistance genes	Colistin MIC and resistance mechanism	Plasmids	Virulence genes
JACWMO000000000	Both (*rmpA2*-5)	pos	K1	O1v2	23	*ybt8*; ICEKp3	*bla*_OXA-232_, *bla*_SHV-11_, *fosA*	MIC 1 µg/ml	IncHI1B (pNDM-MAR) #, ColKP3	*allABDRS*, *fyuA*, *hyi*, *irp1*, *irp2*, *colibactin*, *fdrA*, *gcl*, *glxK*, *glxR*, *aerobactin*, salmochelin, *ybbW*, *ybbY*, *ylbE*, *ylbF*, *mrkABCDFHIJ*
MNPB00000000	*rmpA2* (2)	neg	K24	O2v1	11	*ybt16*; ICEKp12	*RmtF*, *aph(3″)-Ib*, *aph(6)-Id*, *aac(6′)-Ib-cr*, *bla*_SHV-11_, *bla*_TEM-1B_, *bla*_CTX-M-15_, *bla*_OXA-232_, *qnrB1*, *sul2*, *ARR-2*, *fosA*	D150H, T246A, R256G in *pmrB* (8 µg/ml)	IncFIIK, IncFIB(pQil), IncHI1B (pNDM-MAR) #, ColKP3, IncR	*fyuA*, *irp1*, *irp2*, *mrkABCDFHIJ*, aerobactin
MNPC00000000	*rmpA2* (4)	neg	K24	O2v1	11	*ybt16*; ICEKp12	*RmtF*, *aph(3″)-Ib*, *aph(6)-Id*, *aac(6′)-Ib-cr*, *bla*_SHV-11_, *bla*_TEM-1B_, *bla*_CTX-M-15_, *bla*_OXA-232_, *qnrB1*, *sul2*, *ARR-2*, *fosA*	D150H, T246A, R256G in *pmrB* (8 µg/ml)	IncFIIK, IncFIB(pQil), IncHI1B (pNDM-MAR) #, ColKP3, IncR	*fyuA*, *irp1*, *irp2*, *mrkABCDFHIJ*, aerobactin
MNPG00000000	rmpA2* (2)	neg	K24	O2v1	11	*ybt16*; ICEKp12	*RmtF*, *aph(3″)-Ib*, *aph(6)-Id*, *aac(6′)-Ib-cr*, *bla*_SHV-187_, *bla*_TEM-1B_, *bla*_CTX-M-15_, *bla*_OXA-232_, *qnrB1*, *sul2*, *ARR-2*, *fosA*	D150H, T246A, R256G in *pmrB* (8 µg/ml)	IncFIIK, IncFIB(pQil), IncHI1B (pNDM-MAR) #, ColKP3, IncR	*fyuA*, *mrkABCDFHIJ*, aerobactin
MCFQ00000000	*rmpA2* (2)	neg	K24	O2v1	11	*ybt16*; ICEKp12	*rmtf*, *strA*, *strB*, *aac(6′)-lb-cr*, *bla*_TEM-1B_, *bla*_SHV-11_, *bla*_CTX-M-15_, *bla*_OXA-1_, *bla*_NDM-1_, *aac(6′)-lb-cr*, *QnrB1*, *fosA*, *catB3*, *ARR-2*, *sul2*	MIC ≤ 0.12 µg/ml	IncFIB(pQIl), IncHI1B(pNDM-MAR) #, IncFIIK, IncR	*fyuA*, *irp2*, aerobactin, *mrkABCDFHIJ*
SRS3894081	*rmpA2* (9)	pos	K24	O2v1	11	*ybt16*; ICEKp12	*aadA2*, *aph(6)-Id*, *aph(3″)-Ib*, *aac(6′)-lb-cr*, *bla*_SHV-11_, *bla*_TEM-1B_, *bla*_CTX-M-15_, *bla*_OXA-232_, *bla*_OXA-1_, *bla*_NDM-5_, *qnrB1*, *fosA*, *ermB*, *catB4*, *ARR-2*, *sul1*, *sul2*, *dfrA12*	T246A, R256G in *pmrB* (128 µg/ml)	IncFIB(pQil), IncFII, IncFIIK, IncHI1B (pNDM-MAR) #, ColKP3, IncR	*fyuA*, *irp2*, *mrkABCDFHIJ*, aerobactin
SRS3894067	*rmpA2* (2)	neg	K24	O2v1	11	*ybt16*; ICEKp12	*aph(6)-Id*, *aph(3″)-Ib*, *rmtF*, *aac(6′)-lb-cr*, *bla*_SHV-182_, *bla*_TEM-1B_, *bla*_CTX-M-15_, *qnrB1*, *fosA*, *ARR-2*, *sul2*	P95L, T246A, R256G in *pmrB* (64 µg/ml)	IncHI1B (pNDM-MAR) #, IncFIB(pQil), IncR, IncFIIK	*fyuA*, *irp1*, *irp2*, *mrkABCDFHIJ*, aerobactin
SRS3894082	*rmpA2* (9)	neg	K24	O2v1	11	*ybt16*; ICEKp12	*aadA2*, *aph(6)-Id*, *aph(3″)-Ib*, *aac(6′)-lb-cr*, *bla*_SHV-11_, *bla*_TEM-1B_, *bla*_CTX-M-15_, *bla*_OXA-232_, *bla*_OXA-1_, *bla*_NDM-5_, *qnrB1*, *fosA*, *ermB*, *catB4*, *ARR-2*, *sul1*, *sul2*, *dfrA12*	T246A, R256G in *pmrB* (128 µg/ml)	IncFIB(pQil), IncFII, IncFIIK, IncHI1B (pNDM-MAR)#, ColKP3, IncR	*fyuA*, *irp1*, *mrkABCDFHIJ*, aerobactin
MNPD00000000	rmpA2* (2)	neg	K24	O2v1	3791 (SLV of ST11)	*ybt16*; ICEKp12	*RmtF*, *aph(3″)-Ib*, *aph(6)-Id*, *aac(6′)-Ib-cr*, *bla*_TEM-1B_, *bla*_CTX-M-15_, *qnrB1*, *sul2*, *ARR-2*, *fosA*	frameshifted *phoQ*; D150H, T246A, R256G in *pmrB* (8 µg/ml)	IncFIIK, IncFIB(pQil), IncHI1B (pNDM-MAR) #, IncR	*fyuA*, *irp1*, *irp2*, *mrkABCDFHIJ*, aerobactin
MCFP00000000	*rmpA2* (9)	pos	K30	O1/O2v1	43	*ybt9*, ICEKp3	*aacA4*, *rmtf*, *bla*_TEM-1B_, *bla*_SHV-1_, *bla*_CTX-M-15_, *bla*_OXA-181_, *aac(6′)-lb-cr*, *fosA*, *ARR-2*	MIC 0.25 µg/ml	IncFIB(pQIl), IncHI1B(pNDM-MAR) #, IncFIIK, ColpVC	*fyuA*, *irp1*, *irp2*, *kfuA*, *mrkH*, *mrkI*, aerobactin
JADDTT000000000	rmpA2* (6)	neg	K30	O1v1	43	*ybt9*, ICEKp3	*aac(6)-Ib-cr*, *rmtF*, *bla*_OXA-181_, *bla*_SHV_, *bla*_TEM-1B_, *bla*_CTX-M-15_, *fosA*, *ARR-2*	MIC 0.5µg/ml	IncFIB(pQil), ColpVC, IncFIIK, IncHI1B(pNDM-MAR)#	*fyuA*, *irp1*, *irp2*, aerobactin, *mrkH*, *mrkI*, *kfuABC*
PYUL00000000	rmpA2* (6)	neg	K30	O1v1	43	*ybt9*, ICEKp3	*aadA2*, *armA*, *bla*_SHV-11_, *bla*_TEM-1B_, *bla*_CTX-M-15_, *bla*_OXA-181_, *fosA*, *msrE*, *mphE*, *sul1*, *dfrA12*	A217V in *pmrA*; T246A in *pmrB* (8 µg/ml)	IncHI1B (pNDM-MAR)#, IncFIIK, ColpVC, ColRNAI	*fyuA*, *irp2*, *kfuA*, *kfuB*, *mrkH*, *mrkI*, *aerobactin*
PWAD00000000	rmpA2* (5)	neg	K30	O1v1	43	*ybt9*, ICEKp3	*rmtF*, *aacA4*, *bla*_SHV-11_, *bla*_TEM-1B_, *bla*_CTX-M-15_, *bla*_OXA-181_, *aac(6′)-lb-cr*, *fosA*	A217V in *pmrA*; T246A in *pmrB* (32 µg/ml)	IncFIIK, IncFIB(pQil), IncHI1B (pNDM-MAR) #, ColpVC	*fyuA*, *irp2*, *kfuA*, *kfuB*, *mrkH*, *mrkI*, *aerobactin*
SRS3894071	*rmpA2* (5)	neg	K30	O1v1	43	*ybt9*; ICEKp3	*rmtF*, *aacA4*, *bla*_SHV-11_, *bla*_TEM-1B_, *bla*_CTX-M-15_, *bla*_OXA-181_, *aac(6′)-Ib-cr*, *fosA*, *ARR-2*	G53C, A217V in *pmrA*; T246A in *pmrB* (32 µg/ml)	IncFIIK, IncFIB(pQil), IncHI1B (pNDM-MAR) #, ColpVC	*fyuA*, *irp1*, *irp2*, *kfuA*, *mrkH*, *mrkI*, aerobactin
PWAF00000000	*rmpA2* (5)	neg	K30	O1v1	3790 (SLV of 43)	*ybt* unknown	*rmtF*, *aacA4*, *bla*_SHV-12_, *bla*_TEM-1B_, *bla*_CTX-M-15_, *bla*_OXA-181_, *aac(6′)-lb-cr*, *fosA*, *ARR-2*	*mgrB* deletion; A217V in *pmrA*; T246A in *pmrB* (>32 µg/ml)	IncFIIK, IncFIB(pQil), IncHI1B (pNDM-MAR) #, ColpVC	*fyuA*, *irp2*, *kfuA*, *mrkH*, *mrkI*, *aerobactin*
SRS3894072	*rmpA2* (9)	pos	K112	O1v1	15	*ybt16*; ICEKp12	*rmtF*, *aacA4*, *bla*_SHV-11_, *bla*_CTX-M-15_, *bla*_OXA-232_, *aac(6′)-Ib-cr*, *fosA*, *ARR-2*	(MIC 2 µg/ml)	ColKP3, IncFIB, IncHI1B (pNDM_MAR)#	*fyuA*, *irp2*, *kfuA*, *kfuB*, *mrkABCDFIJ*, aerobactin
SRS3894070	*rmpA2* (9)	pos	K112	O1v1	15	*ybt16*; ICEKp12	*rmtF*, *aacA4*, *aph (6)-Id*, *aph(3″)-Ib*, *bla*_SHV-2_, *bla*_TEM-1B_, *bla*_CTX-M-15_, *bla*_OXA-232_, *aac(6′)-Ib-cr*, *fosA*, *ARR-2*, *mphA*, *dfrA12*, *sul2*	Premature stop at 30th amino acid in *mgrB* (32 µg/ml)	IncFIB, IncHI1B (pNDM-MAR)#, ColKP3, Col440I	*fyuA*, *irp1*, *irp2*, *kfuA*, *kfuB*, *mrkABCDFIJ*, aerobactin
SRS3894077	rmpA2* (3)	neg	K2	O1v1	15	*ybt16*; ICEKp12	*Aac(6′)-lb3*, *aac(3″)-lb*, *aph (6)- Id*, *rmtC*, *rmtB*, *bla*_CMY-6_, *bla*_SHV-12_, *bla*_SFO_, *bla*_NDM-1_, *bla*_CTX-M-15_, *bla*_TEM-1B_, *fosA*, *mphA*, *catA1*, *qnrB1*, *sul1*, *sul2*, *aac(6)-lb-cr*	L62P in *phoQ*; T157P in *pmrB* (32 µg/ml)	IncC, IncFIB(pQil), IncFIB, IncHI1B (pNDM-MAR)#, IncFII	*fyuA*, *irp2*, aerobactin, salmochelin, *mrkABCDFIJ*
SRS3894080	Both* (*rmpA*-2, *rmpA2*- 4)	pos	K2	O1v1	86	*ybt* unknown	*Aac(6′)-Ib-cr*, *AadA1*, *Aph(3′)-VI*, *bla*_SHV-28_, *bla*_TEM-1A_, *bla*_CTX-M-15_, *bla*_OXA-9_, *bla*_NDM-1_, *qnrS1*, *fosA*	Premature stop at 3rd amino acid in *mgrB* (32 µg/ml)	IncFIB(pQil), IncFIIK, IncHI1B (pNDM-MAR) #, ColRNAI	*fyuA*, aerobactin, salmochelin, *kvgA*, *kvgS*, *mrkABCDFHIJ*
JACWMP000000000	Both* (*rmpA*-2, *rmpA2*-2)	pos	K57	O2v2	4847 (TLV of ST29)	Unknown	*bla*_SHV-71_, *bla*_OXA-232_, *bla*_DHA-1_, *qnrB4*, *sul1*, *fosA*	MIC 0.5 µg/ml	ColKP3, IncHI1B (pNDM-MAR) #, IncFIBK, IncFII	*allABDRS*, *fyuA*, *hyi*, *irp1*, *colibactin*, *aerobactin*, *salmochelin*, *ybbW*, *ybbY*, *ylbE*, *ylbF*, *mrkABCDFHIJ*
MCFO00000000	Absent	neg	K51	O2v2	231	*ybt14*, ICEKp5	*aadA2*, *aacA4*, *bla*_TEM-1B_, *bla*_SHV-1_, *bla*_CTX-M-15_, *bla*_OXA-232_, *mphA*, *ermB fosA*, *aac(6′)-lb-cr*, *ARR-2*, *sul1*, *catA1*, *dfrA12*	MIC 0.5 µg/ml	IncFIA, IncFIB, ColKp3, IncFIIK, IncFII	*fyuA*, *kfuA*, *kfuB*, aerobactin, salmochelin, *mrkABCDFHIJ*
SGIY00000000	Absent	neg	K51	O1v2	231	*ybt14*; ICEKp5	*aadA2*, *aac(6′)-Ib3*, *rmtF*, *aac(6′)-lb-cr*, *bla*_SHV-28_, *bla*_TEM-1B_, *bla*_CTX-M-15_, *bla*_OXA232_, *fosA*, *mphA*, *ermB*, *qnrS1*, *catA1*, *ARR-2*, *sul1*, *dfrA12*	Premature stop codon at the 9th amino acid in *mgrB* (>4 µg/ml)	ColKP3, IncFIA, IncFIB, IncFIB(pQil), IncFII, IncFIIK	*fyuA*, *irp1*, *irp2*, *kfuA*, *kfuB*, *mrkBCDFHIJ*, aerobactin
JAAEYE000000000	Absent	neg	K51	O1v2	231	*ybt14*; ICEKp5	*aadA2*, *aac(6′)-Ib3*, *aac(6′)-lb-cr*, *bla*_SHV-28_, *bla*_TEM-1B_, *bla*_CTX-M-15_, *bla*_OXA-232_, *fosA*, *mphA*, *ermB*, *catA1*, *ARR-2*, *sul1*, *dfrA12*	MIC ≤ 1 µg/ml	ColKP3, IncFIA, IncFIB(pQil), IncFII, IncFIIK	*fyuA*, *irp1*, *irp2*, *kfuA*, *kfuB*, *mrkBCDFHIJ*, aerobactin
JAHPLH000000000	Absent	neg	K51	O1v2	231	*ybt14*; ICEKp5	*aadA2*, *rmtF*, *bla*_SHV-1_, *bla*_TEM-1D_, *bla*_OXA-232_, *mphA*, *ermB*, *sul1*, *qnrS1*, *dfrA12*	MIC ≤ 0.25 µg/ml	ColKP3, IncFIA, IncFIB(pQil), IncFIIK, IncFII	*fyuA, irp1, irp2*, aerobactin, salmochelin, *mrkABCDFHIJ*
JAHPLI000000000	Absent	neg	K51	O1v2	231	*ybt14*; ICEKp5	*rmtF*, *bla*_SHV-1_, *bla*_TEM-1D_, *bla*_OXA-232_, *bla*_CTX-M-15_, *mphA*, *ermB*, *sul1*, *qnrS1*, *ARR-2*	MIC ≤ 0.5 µg/ml	ColKP3, IncFIA, IncFIB(pQil), IncFIIK, ColRNAI	*fyuA, irp1, irp2*, aerobactin, *kfuABC, mrkBCDFHIJ*
JAARNY000000000	Absent	neg	K51	O1v2	231	*ybt14*; ICEKp5	*aadA2*, *aac(6′)-lb*, *bla*_SHV-1_, *bla*_TEM-1D_, *bla*_OXA-232_, *bla*_CTX-M-15_, *mphA*, *ermB*, *sul1*, *qnrS1*, *ARR-2*	MIC 0.5 µg/ml	ColKP3, IncFIA, IncFIB(pQil), IncFIIK, Col440I	*fyuA*, *iroB*, *irp1*, *irp2*, *kfuABC*, aerobactin, *clbA*, *mrkABCDFHIJ*

Colistin resistance amino acid substitutions: P, proline; L, leucine; T, threonine; A, alanine; R, arginine; G, glycine; D, aspartic acid; V, valine; ybt, Yersiniabactin.

ST, sequence type; SLV, single-locus variant; TLV, triple-locus variant; MIC, minimum inhibitory concentration; Kp, Klebsiella pneumoniae.

*Frameshift mutation was observed in rmpA2; variants of rmpA and rmpA2 are mentioned in brackets.

Virulence plasmid.

^Mutation in rmpA.

Among the study population, 16 patients developed hospital-acquired CR-hvKp infection, while three acquired community-associated infection. The nature of infection in the other eight patients could not be established from electronic medical records. A combination of colistin and tigecycline was most frequently used to treat these CR-hvKp infections (n = 11, 44%). The other antimicrobials that were used in combination therapy include meropenem with colistin/tigecycline and metronidazole. Twelve patients with CR-hvKp had a fatal outcome, and the mean duration of stay in the hospital was 91 days among this cohort. Besides, half of the study population were neutropenic and immunosuppressed.

### Genomic Characterization of Carbapenem Resistant Hypervirulent *Klebsiella pneumoniae*

The genomic characteristics of the CR-hvKp are detailed in **Table 1**. The virulence plasmid present in the isolates predominantly carried IncHI1B (pNDM-MAR) backbone. As shown in **Table 1**, most of the CR-hvKp belonged to international high-risk clones such as ST11, ST15, and ST43, which carry ARGs on multiple plasmids.

Interestingly, there was a single CR-hvKp belonging to ST23 that carried both *rmpA* and *rmpA2* on the virulence plasmid. In addition, it carried the carbapenemase-encoding gene, *bla*_OXA-232_, on ColKP3 plasmid. When compared with other study isolates, notably, this isolate carried additional virulence factors such as colibactin, salmochelin, and those for allantoin metabolism (*hyi*, *glxK*, *glxR*, *ybbW*, *ybbY*, *ylbE*, and *ylbF*). The other isolates are briefly described below and carry diverse K and O antigens depending on the clonal group they belong to.

#### CG11

Eight isolates belonged to CG11, of which seven belonged to ST11, while a single isolate was of ST3791, a single-locus variant of ST11. All the isolates of CG11 belonged to capsule type, K24, with O2v1 O antigen. These isolates coded for yersiniabactin (*ybt16*) mobilized by ICEKp12. The isolates lacked *rmpA* but carried *rmpA2*. Isolates of CG11 carried *bla*_NDM_ and *bla*_OXA-232_ alone or in combination. Seven isolates in this group were resistant to colistin and carried two amino acid substitutions in *pmrB* (T246A and R256G) as shown in **Table 1**. The other mutations conferring colistin resistance include frameshift in *phoQ* in the isolate belonging to ST3791.

#### CG43

Five isolates belonging to ST43 and one of ST3790, a single-locus variant of ST43, were identified. All the six isolates carried a frameshifted *rmpA2* in the virulence plasmid. ST43 isolates carried *ybt-9* on ICEKp3, while the isolate belonging to ST3790 carried *ybt* of unknown type. The isolates belonged to K30 with O1v1 antigen. Isolates of CG43 lacked *bla*_NDM_ and predominantly carried *bla*_OXA-181_, except one isolate that encoded *bla*_OXA-232_. Three isolates belonging to ST43 were resistant to colistin due to single amino acid substitutions in *pmrA* (A217V) and *pmrB* (T246A) as mentioned in **Table 1**. *K. pneumoniae* ST3790, in addition to the mutations in *pmrA* and *pmrB*, showed deletion of *mgrB*, which confers resistance to colistin.

#### ST231

**Figure 1:**
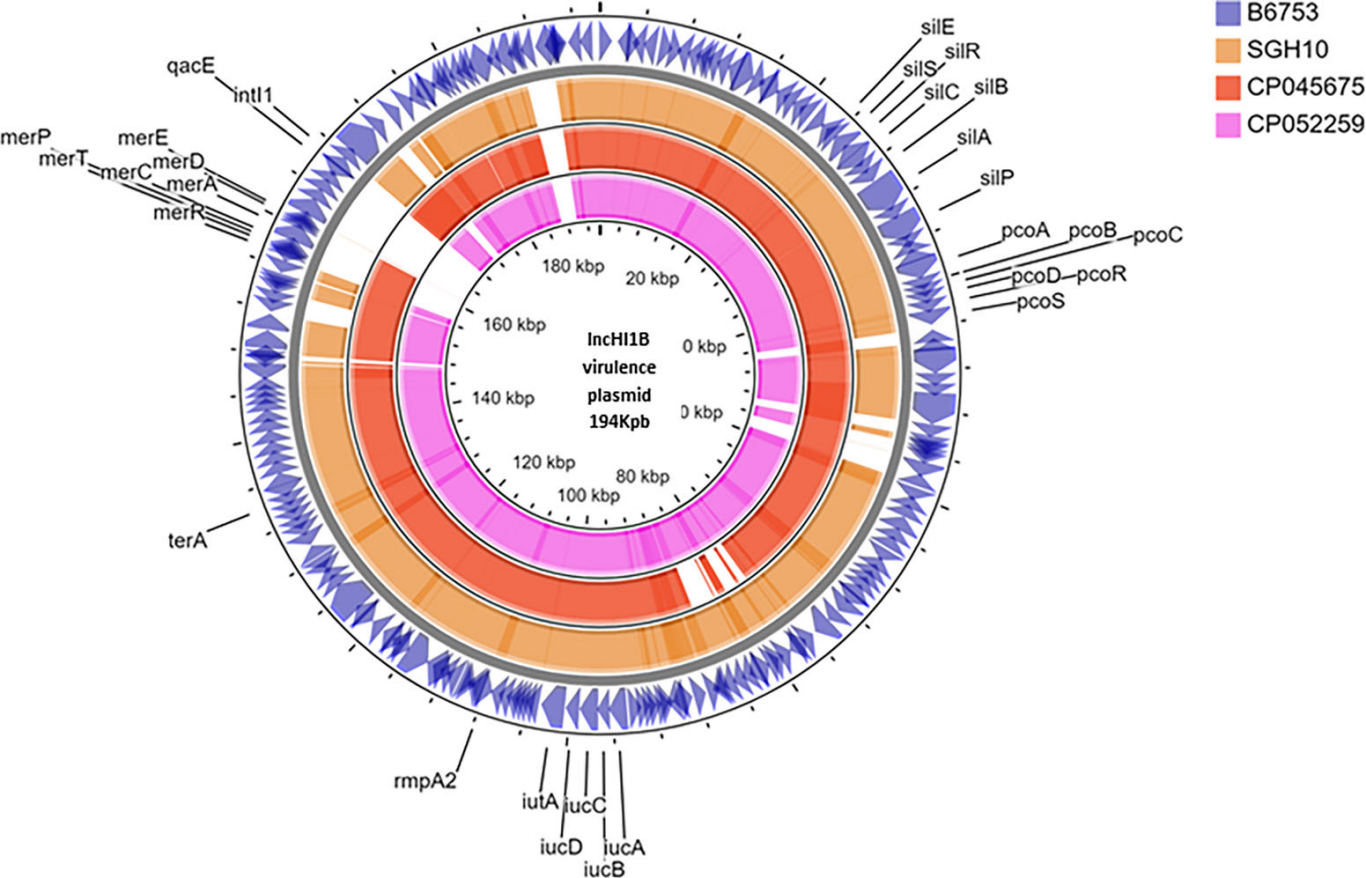
Circular representation of virulence plasmid assembled from Kp B6753 displayed using CG view server. Comparison of virulence plasmid carrying *rmpA2* among (1) Kp strain B6753 (CP067046) from ST43 (2) Kp strain SGH10 (CP025080) from ST23 (3) Kp strain WSD411 (CP045675) from ST15 (4) Kp strain E16KP0290 (CP052259) from ST65.

Six isolates belonging to ST231, although lacked the *rmpA2*, carried aerobactin, one of the key virulence factors. In two isolates, aerobactin was identified on a plasmid with IncFIA-IncFII replicons (data not shown). However, for the other isolates since complete genomes were not available, it was impossible to determine if aerobactin was inserted into chromosome or another plasmid excluding the virulence plasmid. These isolates carried *bla*_OXA-232_ mobilized by ColKP3 plasmid. **Figure 1** shows the virulence plasmid (carrying aerobactin) in isolate B6753 (CP067046) compared with the reference virulence plasmid SGH10 (CP025080) and the closest-matching plasmids CP045675 and CP052259. The virulence plasmid of B6753 carries integron class 1, which is absent in the other three plasmids. Heavy metal resistance genes such as those coding for copper (*pco*), silver (*sil*), tellurium (*ter*), and mercury (*mer*) are also shown in **Figure 1**. A single isolate in this group was non-susceptible to colistin due to truncated *mgrB* of eight amino acids (**Table 1**).

**Figure 2:**
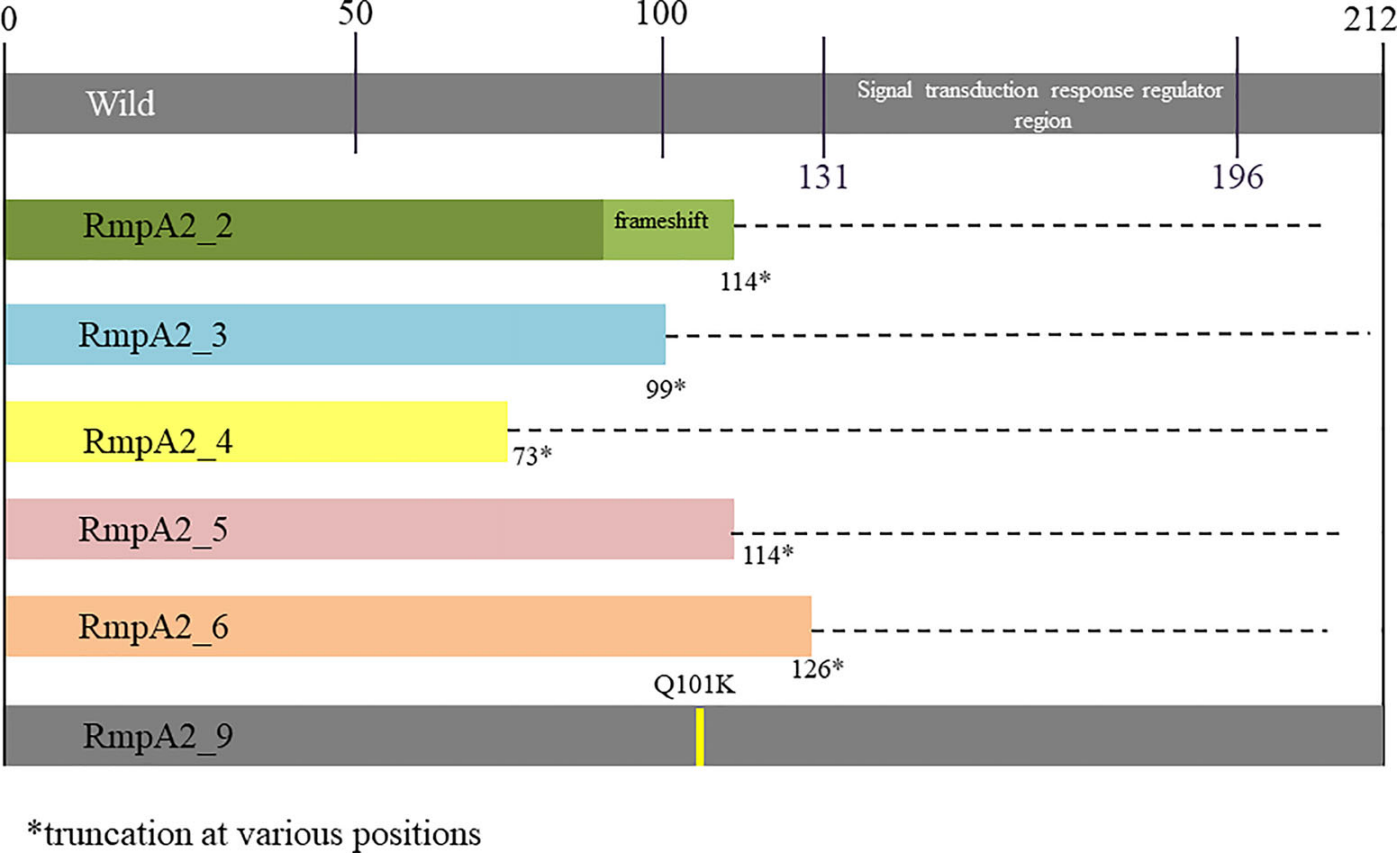
Variants of RmpA2 observed among carbapenem resistant hypervirulent Kp from India..

#### Other Clones

Three CR-hvKp belonged to ST15, while a single isolate each belonged to ST86 and ST4847 (TLV of ST29). Two isolates of ST15 were non-susceptible to colistin but showed different chromosomal mutations (**Table 1**). Interestingly, an isolate belonging to ST15 carried the K2 capsule type, which is in contrast to the present knowledge of K2 antigen being confined to hvKp clones such as ST65 and ST86. This isolate also showed the presence of frameshift mutation in *rmpA2*. The isolate belonging to ST86 was associated with K2 antigen, a characteristic feature of hvKp, which is also reported by other studies. ST86 *K. pneumoniae* in this study also carried non-functional *mgrB* due to premature stop codon in the third amino acid.

Among the other factors contributing to virulence, the siderophore salmochelin was present in a single isolate each of ST15, ST86, ST231, and ST4847. Interestingly, the CRKp in the present study have acquired the virulence plasmid with *rmpA2*, while *rmpA* was present in three isolates only. In addition, aerobactin, present in all the isolates, was the other promising molecular marker among these isolates.

### RmpA2 Variants

Among the study isolates, six *rmpA2* variants were identified as shown in **Figure 2**. Variants 2–6 were truncated at various positions, while *rmpA2*_9 had a single amino acid substitution at position 101 wherein glutamine is replaced by lysine. The wild-type *rmpA2*, encoded by 212 amino acids, has the signal transduction response regulator region from 131 to 196 amino acids. This region is disrupted in the variants leading to a functional loss, which reflects as a lack of hypermucoviscous phenotype. Hence, the CR-hvKp cannot be screened using a positive string test.

**Figure 3:**
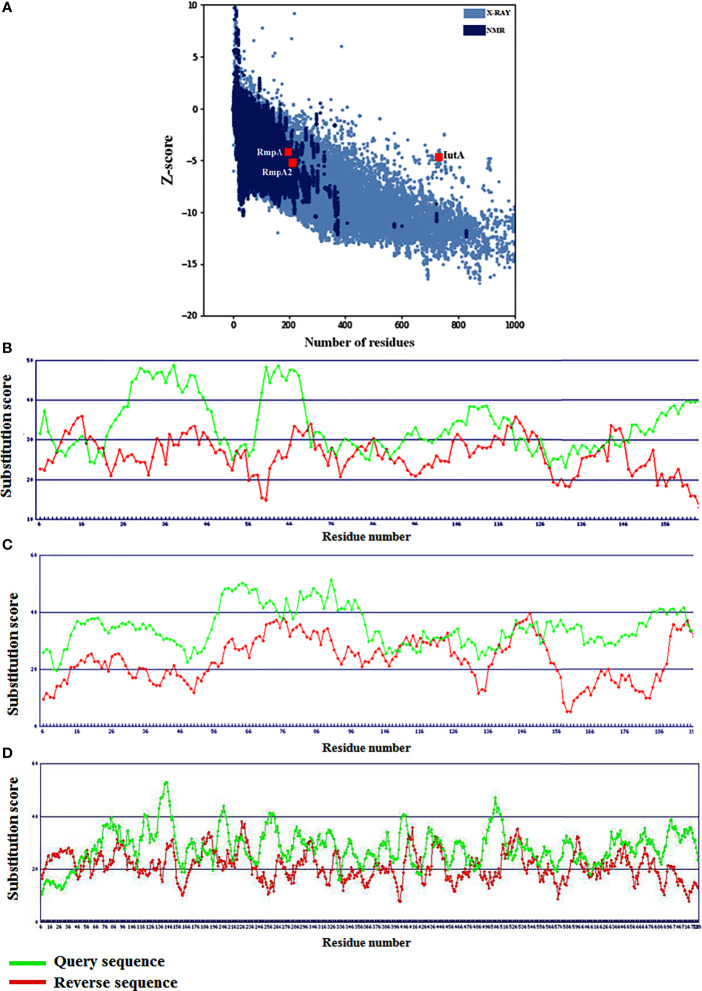
Global model qualities of RmpA, RmpA2 and IutA with respect to **(A)** experimentally derived structures and **(B–D)** residue-level substitution scores signifying local folding conformations.

### Protein Modeling and Structural Validations

Based on Z-scores from ProSA-web signifying the overall quality of the modeled RmpA, RmpA2, and IutA proteins, it was observed that all three are well-poised among experimentally determined (NMR and X-ray diffraction) protein structures (**Figure 3A**). The residue-level substitution scores of the validated structures of RmpA, RmpA2, and IutA proteins as per the HARMONY calibration plot are considerably higher than the respective reverse sequences (control) designating minimum errors in terms of misfolded conformations (**Figures 3B–D**). After final refinement, the modeled structures possessed >95% residues in Ramachandran favored region with low values corresponding to poor rotamers (<1.5), which signify the conformational integrity of the protein structures.

**Figure 4:**
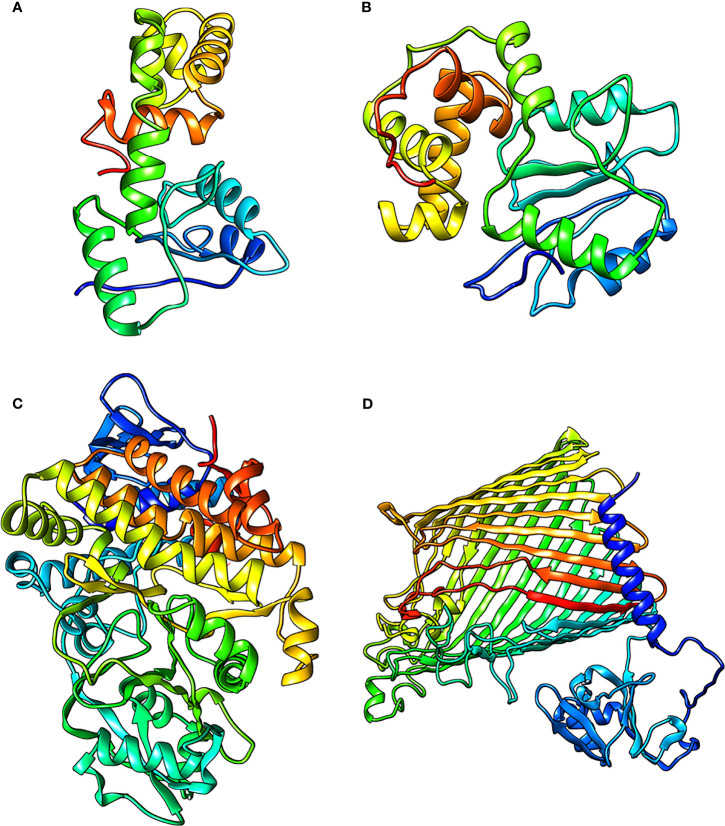
The refined structures of the proteins **(A)** RmpA **(B)** RmpA2 **(C)** IucA and **(D)** IutA.

The final predicted structures of RmpA, RmpA2, and IutA were submitted to Protein Model Database (PMDB) (http://srv00.recas.ba.infn.it/PMDB/) having PMDB-IDs PM0083529, PM0084102, and PM0084103. The final structures of all the classical proteins are shown in **Figures 4A–D**.

**Figure 5:**
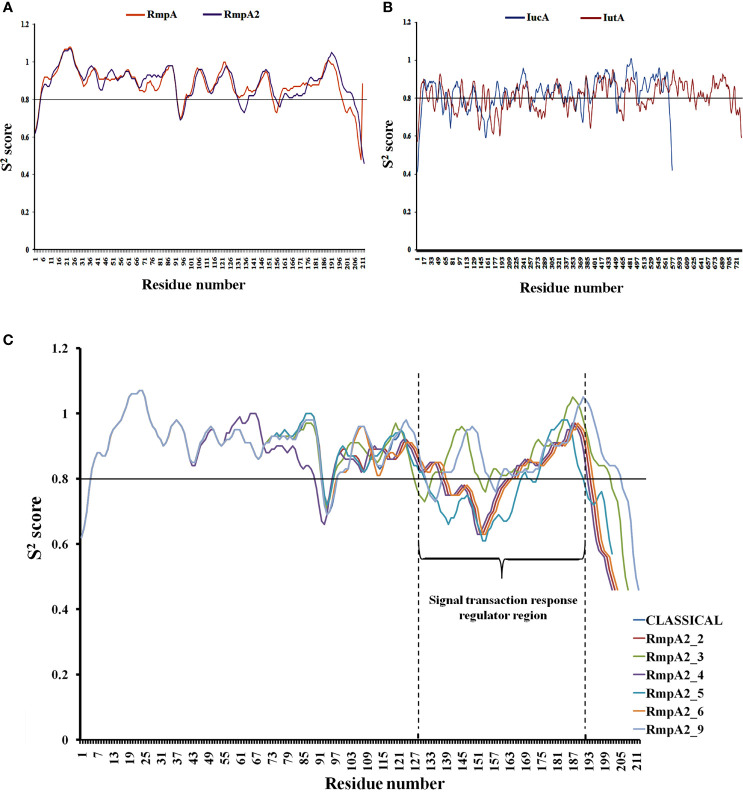
Backbone stability analysis: The relatively rigid (S2 score>0.8) and flexible (S2 score<0.8) regions of **(A)** RmpA and RmpA2 **(B)** IutA and IucA. **(C)** The variants of RmpA2 with shifts in the backbone as a resultant of frameshift mutations.

### Backbone Stability of the Proteins

**Figures 5A, B** show that all the proteins have a stable backbone conformation [high average rigidity of backbone ➔ S^2^ > 0.8], conferring structural integrity to the proteins.

**Figure 6:**
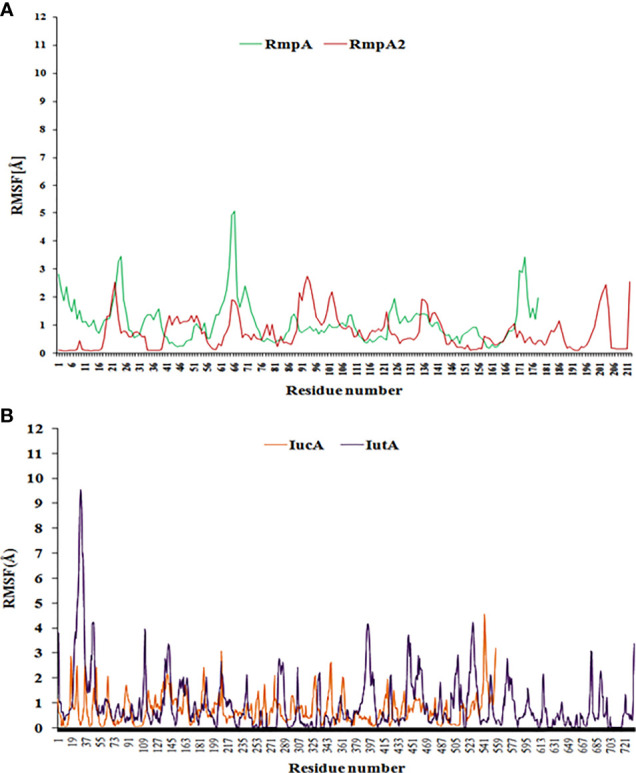
The RMSF curve from coarse-dynamics simulations. Relative RMSF of **(A)** RmpA and RmpA2 **(B)** IucA and IutA.

The functionally significant domains of IucA are aerobactin-siderophore biosynthesis domain (between residues 138 and 361, average S^2^ score = 0.80) and ferric-ion reductase domain (between residues 400 and 556; average S^2^ score = 0.88). The characteristic membrane TonB-dependent receptor domain of IutA is located between residues 238 and 730 (average S^2^ score = 0.82), and a smaller plug domain is located between residues 45 and 146 (average S^2^ score = 0.79). The signal transduction regulator domain (STRD) of RmpA2 is located between amino acid residues 131 and 196 (average S^2^ score = 0.87). The average S^2^ scores indicate that both IutA and IucA have high backbone rigidity in their functional domains as well as the entire structure [overall 0.80 ± 0.02 Å]. Hence, they have stable structure-function profiles. The overall backbone rigidity of RmpA2 (0.88 ± 0.006 Å) is marginally higher than that of IutA and IucA. Hence, classical RmpA2 has better backbone stability, which supported its previous consideration as a molecular marker for hvKp (Russo et al., 2018). However, owing to indel mutations in our isolates between nucleotides 285 and 290, major frameshifts were observed in the backbone trajectory of RmpA2 variants (**Figure 5C**) when compared with the classical RmpA2. The mutant RmpA2_3 showed identical trajectory when compared with the classical counterpart, although there was an upstream shift of the STRD. The mutant RmpA2_4 portrayed a slightly different frameshift than the rest owing to an insertion mutation at nucleotide position 197.

All of the mutants of RmpA2 were observed to have major reductions S^2^ scores, especially in the SRTD domain (by ~40%) converting the structural profile of this region from a stably rigid domain to a considerably flexible one owing to upstream truncations. These changes can potentially contribute to the under-functioning of RmpA2 in the variants, thereby failing to express hypermucoviscous phenotype. However, no such truncations/indel mutations were observed in IutA or IucA, and they expressed a uniformly stable profile in all the isolates.

### Coarse Dynamics Analysis to Assess the Structural Stability Profile

The relative stability of RmpA, RmpA2, IutA, and IucA analyzed using CABSflex server showed that that the relative fluctuations of RmpA are higher than those of RmpA2 (**Figure 6A**), although IutA and IucA possessed similar dynamicity (**Figure 6B**). From the conformational perspective, it can be inferred that classical RmpA2 possesses a better stability profile than RmpA as well as IucA and IutA, which supports the backbone dynamicity of the proteins. Furthermore, the average RMSF of the residues in signal regulatory region of RmpA2 (residues between 131 and 196) is lower (0.59 Å) than the average RMSF of the transcription regulatory residues (residues between 109 and 156) in RmpA (0.91 Å). This portrays a better structure-function stability profile of RmpA2 over RmpA and hence is predominantly present among CR-hvKp. The average RMSF of the functional domain residues in aerobactin such as the aerobactin-synthetase domain (IucA) and ferric-ion reductase (IutA) domain is 0.79 and 0.77 Å, respectively. The receptor domain (residues 338–730) of IutA has fairly lower fluctuations (average RMSF = 0.77 Å), similar to IucA. This portrays a better structure-function stability profile of aerobactin proteins over RmpA, but not RmpA2. However, in truncated RmpA2 proteins, the resultant RMSF values are very high (and irregular), which do not follow a pattern as compared with the parent RmpA2 protein (not shown).

**Figure 7:**
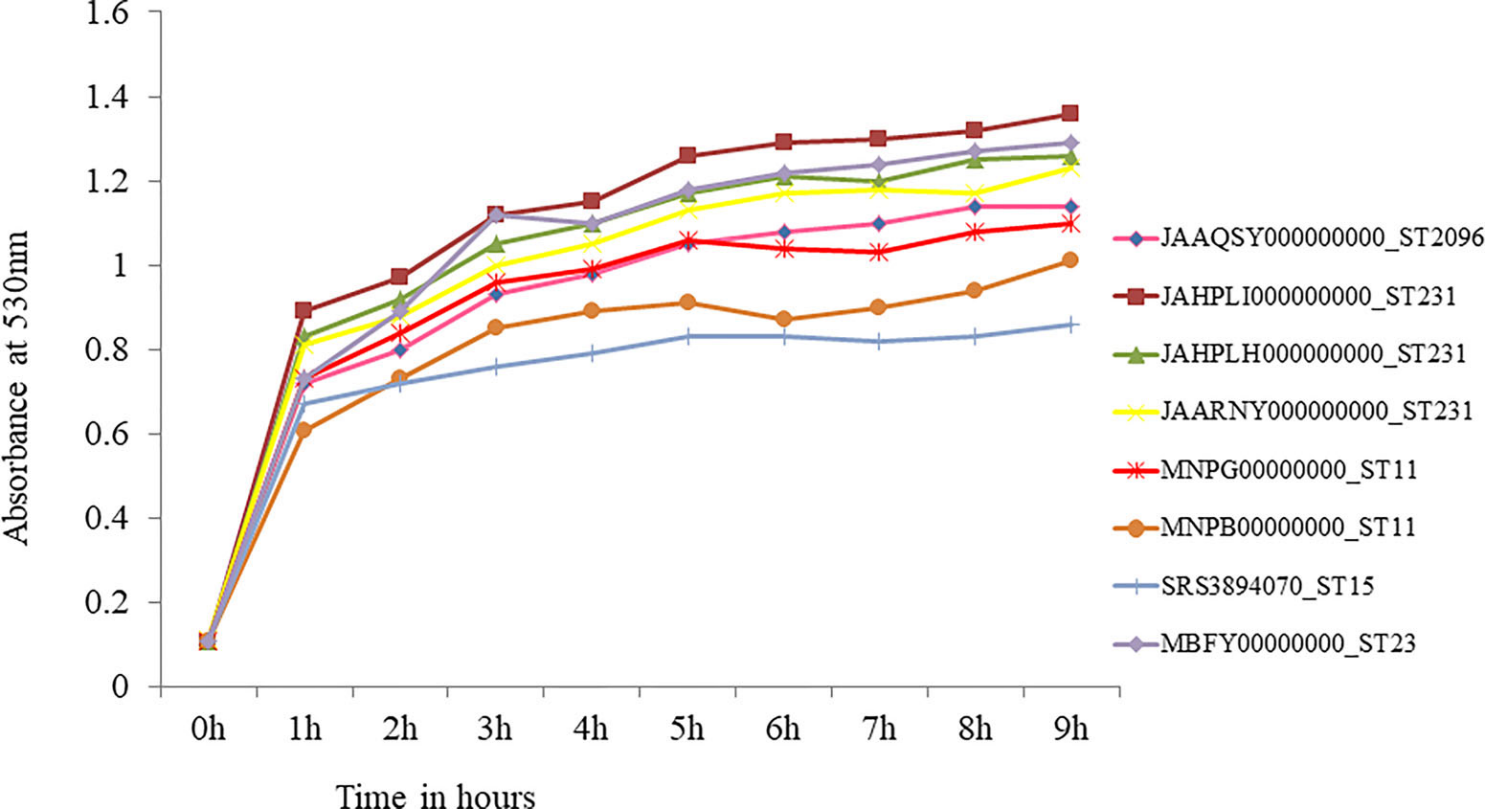
*In-vitro* growth curve of hypervirulent *Klebsiella pneumoniae*. Isolate belonging to ST23 (MBFY00000000) was pan-susceptible and carries wild type *rmpA* and *rmpA2*. Isolates belonging to ST231 (JAHPLI000000000, JAHPLH000000000, JAARNY000000000) that carried aerobactin alone showed similar growth when compared to ST23. CRKp belonging to high risk clones ST11 (*rmpA2*_2, MNPG00000000; *rmpA2*_2, MNPB00000000) and ST15 (*rmpA2*_9; SRS3894070) showed slower growth than ST23 and ST231 hvKp.

### *In-Vitro* Fitness of Hypervirulent *Klebsiella pneumoniae*

Growth curve was obtained for eight hypervirulent *K. pneumoniae*, which is shown in **Figure 7**. An isolate belonging to ST2096, carrying truncated *rmpA2* (JAAQSY000000000), and an isolate with wild-type *rmpA2* belonging to ST23 (MBFY00000000) isolated at the study center were used to compare the growth rate of other CR-hvKp in the present study. Three isolates carrying aerobactin without *rmpA*/*rmpA2* belonging to ST231 showed a similar growth rate as compared with a pan-susceptible ST23 isolate that carried wild-type *rmpA* and *rmpA2*. The isolates belonging to high-risk clones ST11 and ST15 with truncated *rmpA2* grew slower than ST23 and other isolates carrying aerobactin alone (ST231) as shown in **Figure 7**. ST11 MDR-hvKp carried *rmpA2*_2 truncated to 114 amino acids, while ST15 MDR-hvKp carried *rmpA2*_9 with a single amino acid substitution. However, these findings need to be confirmed in animal models to establish the virulence of strains carrying aerobactin alone. In addition to virulence factors, the role of plasmids carrying antimicrobial resistance in impelling the growth rate among these isolates needs further validation.

### Correlation of *In-Vitro* and *In-Silico* Results

The absence of *rmpA* from the virulence plasmid of most CR-hvKp in the present study is warranted by the least structural stability of RmpA deduced from *in silico* analysis. Artemis comparison tool (Carver et al., 2005) was used to compare the plasmid compositions of the study isolate (CP067046) with reference virulence plasmid, SGH10 (CP025081) and pE16KP0290-1 (CP052259). Feature statistics showed that the relative guanine–cytosine (GC) content (%) of *rmpA* (<35%) is significantly lower than that of aerobactin genes *iucA* and *iutA* (>55%). Higher GC content not only depicts higher genomic stability but also projects an enhanced translation process and efficient amino acid usage (Li et al., 2015) of aerobactin over *rmpA*. Among CR-hvKp, there is additional fitness cost when compared with CRKp or hvKp due to the presence of both resistance and virulence plasmids. Furthermore, the presence of unstable proteins imposes additional energy expenditure by the organism in maintaining the protein, which can be balanced by losing the protein. The frameshifted *rmpA2* with functional aerobactin is retained on the virulence plasmid taken up by the CRKp clones. As described in the previous section, aerobactin has lower fluctuations in the receptor domain with overall high backbone stability and hence has not undergone functional changes like RmpA2. Interestingly, in our experience, apart from the endemic clone ST231, other classical MDR *K. pneumoniae* that lacked a virulence plasmid and belong to diverse clones such as ST11, ST14, and ST147 did not carry aerobactin (data not shown; genomes available in PRJNA400267). Therefore, it can be inferred that the CR-hvKp has reserved aerobactin, an important virulence trait, and is prone to eliminate *rmpA* and *rmpA2*.

## Discussion

Hypervirulent Kp is the most vicious pathotype of Kp that is now seen carrying antimicrobial resistance unlike its earlier counterpart (Chen et al., 2020; Zhang et al., 2020; Yang et al., 2021). Though in the last decade the virulent clones and MDR clones of Kp were distinct, the present decade has witnessed the convergence of these two groups, leading to MDR-hvKp. The formation of MDR-hvKp can be elucidated in three ways: a) acquisition of a virulence plasmid by MDR clones, b) acquisition of AMR plasmids by the virulent clones, and c) formation of mosaic plasmids that carry two replicons and code for both antimicrobial resistance and virulence (Tang et al., 2020). To the best of our knowledge, this is the first report describing the structures of RmpA, RmpA2 and variants of RmpA2 among CR-hvKp belonging to high-risk international clones such as ST11, ST15, and ST231 from the Indian subcontinent. Here, the mechanism of CR-hvKp formation is due to the acquisition of a virulence plasmid by CRKp clones.

The key virulence factors among hvKp include the siderophore aerobactin, and other genes such as *rmpA*, *rmpA2*, and *peg-344*, which are borne on the virulence plasmid. pLVPK, a 200-kbp plasmid, a prototype of Kp virulence plasmid, was first identified in isolate CG43; and the loss of this plasmid conferred absence of hypermucoid colonies (Lai et al., 2003). Over the years, several screening and confirmatory methods have been used to identify hvKp, which include the string test, determining the virulence in animal/moth models and PCR detection of virulence genes (Shon et al., 2013; Russo and MacDonald, 2020). *G. mellonella* killing assay used in conjugation with the string test is a relatively simple and accurate method to assess Kp virulence and differentiate between hvKp and cKp (Russo and MacDonald, 2020). In the present study, string test followed by detection of *rmpA, rmpA2* and aerobactin was used to define hvKp. Poor sensitivity and specificity of string test have been demonstrated earlier among ESBL-hvKp, similar to the present study results (Yu et al., 2015). The study reported absence of *rmpA* and mutations in *rmpA2* among ESBL-hvKp in addition to lack of hypermucoviscous phenotype, similar to the observations among CR-hvKp in the present study. Hence, it is evident that in the presence of AMR genes, *rmpA* is lost while *rmpA2* is non-functional due to fitness cost.

The capsule polysaccharide production has three transcription units (*orf1-2*, *orf3-15*, and *orf16-17*) in Kp, among which two are directly influenced by *rmpA*, while one is affected by *rmpA2*. The deletion of *rmpA* and *rmpA2* leads to decreased capsular polysaccharide under anaerobic conditions (Lin et al., 2019), which is also observed in the present study. Hence, CR-hvKp that lacks *rmpA* and *rmpA2* lacks hypermucoviscous phenotype due to decreased capsular polysaccharide production.

In accordance with the speculations that *rmpA* is lost among CR-hvKp, we modeled the proteins RmpA and RmpA2 to determine the structural stability. It was observed that RmpA2 has a better stability profile than RmpA; and hence in MDR bacteria, RmpA2 is retained. Among the CR-hvKp, not only is the virulence plasmid losing *rmpA*, but there is also the formation of a mosaic plasmid that carries *rmpA2* in addition to the ARGs. These events can be explained by the *in silico* results. It is noteworthy that the mosaic plasmids reported from our hospital earlier carried *rmpA2* alone among CR-hvKp belonging to ST2096 (Shankar et al., 2020b). Hence, among CR-hvKp, string test is obsolete; and though molecular detection of *rmpA2* can be performed, it is essential to determine the completeness of *rmpA2* for gaining insights into the evolution of CR-hvKp.

In view of this, the stability profile of aerobactin-encoding proteins IucA and IutA was investigated. Aerobactin-encoding proteins had uniform high backbone rigidity and lower conformational fluctuations especially in the functional domains as compared with RmpA2 variants and hence is less prone to structural instability. Therefore, we hypothesize that CR-hvKp will retain functional aerobactin and exploit it as the key virulence factor. This is especially beneficial during the formation of mosaic plasmids that carry both virulence genes and ARGs, as it reduces the expenditure of *rmpA2* expression. In addition, several studies have demonstrated the prerequisite of aerobactin for survival of hvKp in animal models, and cKp lack aerobactin, while they may carry other incompetent siderophores such as enterobactin and yersiniabactin (Russo et al., 2015). Aerobactin has also been identified as an ideal anti-virulence target since it has the added advantage of being unaffected by antimicrobial resistance of the hvKp (Russo and Gulick, 2019). **Table 2** lists the key virulence factors that are observed among various resistant and virulent pathotypes of Kp deduced from previous reports and observations. **Table 3** lists the virulence factors of hvKp along with their function and location in hvKp genome.

**Table 2:** Key virulence markers among various pathotypes of Kp.

	Classical Kp (cKp)	Hypermucoviscous Kp (hmKp)	Hypervirulent (hvKp) susceptible Kp	Hypermucoviscous hypervirulent susceptible Kp	Hypervirulent multidrug-resistant Kp
**String test**	−	+	+/−	+	−
**Virulence plasmid**	−	+/−	+	+	+
**Mosaic plasmid**	−	−	−	−	+
**Wild-type *rmpA***	−	v	+ (100%)	+ (100%)	− (95%)
**Wild-type *rmpA2***	−	v	+ (100%)	+ (100%)	−
**Mutated *rmpA2***	−	−	−	−	+ (100%)
**Aerobactin**	−	−	+ (100%)	+ (100%)	+ (100%)
**Salmochelin**	−	−	+/− (80%)	+/− (80%)	+/− (<10%)
**Peg-344**	−	−	+ (v)	+ (v)	+/− (v)
**Phenotypic identification**	Identified	Identified	Identified	Identified	unidentified
**Genotypic identification**	Not essential to designate as cKp	Not essential to designate as hmKp	Mandatory to identify as hvKp	Mandatory to identify as hvKp	Mandatory to identify as hvKp
**Correlation with clinical features**	Not essential to designate as cKp	Not essential to designate as hmKp	Mandatory to identify as hvKp	Mandatory to identify as hvKp	Mandatory to identify as hvKp
**Clones**	Diverse	Diverse	CC23, CC65	CC23, CC65	Regional, driven by resistance profile
**Clinical impact**	Less pathogenic; good clinical outcome; Treatment with antibiotics	Challenges antimicrobial penetration though less virulent	Fatal outcome though susceptible Good biofilm formers	Fatal outcome; poor antibiotic penetration Good biofilm formers	Fatal outcome

Kp, Klebsiella pneumoniae; V, variable; CC, clonal complex.

Globally, there is a surge in the international high-risk clones acquiring virulence factors among Kp. Several studies on the acquisition of a virulence plasmid by ST11 KPC-producing Kp are increasingly reported from China, where it is endemic (Hu et al., 2021). Hu and colleagues report the uptake of a virulence plasmid among *bla*_KPC-2_ carrying ST11 Kp from various regions in China. The IncHI1B virulence plasmid in these isolates carried *rmpA* and *rmpA2*, or *rmpA2* alone. Interestingly, two clades of ST11 have been identified in China, which carries different capsule types: clade1 with K64 capsule type and clade2 with K47 capsule type (Hu et al., 2021). Clade1 was associated with increased virulence factors when compared with clade2. Furthermore, Li and colleagues report mosaic plasmids with IncHI1B–IncFIB replicons carrying virulence gene *rmpA2* and aerobactin among ST11 KPC Kp in China (Li et al., 2021). Notably, in the present study, CR-hvKp belonging to ST231 lacked *rmpA* and *rmpA2*, while it encoded aerobactin. In the present study setting, ST231 CRKp is the endemic clone predominantly carrying *bla*_OXA-232_ on ColKP3 plasmid (Shankar et al., 2021). As speculated, for better survival of this endemic clone, aerobactin has been acquired while excluding *rmpA* and *rmpA2*. This is another possible evolutionary mechanism of the endemic clones acquiring a portion of the virulence plasmid that is of utmost importance while compromising on the other regions of the virulence plasmid. We hypothesize that the aerobactin might be incorporated into the chromosome of endemic clones such as ST231, which will further lead to the establishment of virulent endemic clones, causing nosocomial outbreaks.

One of the most important characteristics used for identification of hvKp include the clinical presentation of the patient. In a previous study at our center, community-acquired hvKp infection showed a fatal outcome when compared with HAIs; however, the study was limited by a small sample size (Shankar et al., 2018). In the present study, a mortality of 46% can be attributed to the CR-hvKp that belong to HAI clones and hence were challenging to treat due to the limited susceptibility to antimicrobials. Interestingly, in the study cohort, there was no case of liver abscess, which explains the absence of classical hvKp presentation. It is well known that diabetes mellitus and Asian ethnicity predispose to hvKp infections (Choby et al., 2020). Mortality among hvKp-infected patients range from 30% to 100% as reported in various studies and is also due to the antimicrobial susceptibility of the infecting hvKp (Choby et al., 2020). Unfortunately, there are no specific therapeutic choices targeting hvKp, and discrete use of antimicrobials based on susceptibility testing must be employed. In cases of localized infection with hvKp, source removal is the most important technique in infection management (Shon et al., 2013).

Though MDR-hvKp and CR-hvKp are being increasingly reported, there is a lack of rapid test to identify this pathotype. Future prospects include the development of immunochromatographic assays that can be deployed for rapid identification of hvKp, in which cultures of *K. pneumoniae* can be used to determine the presence of *rmpA*, *rmpA2*, and aerobactin, especially among carbapenem-resistant isolates. This test can be used to target the signal transduction region in these proteins, which is disrupted in the *rmpA2* mutant hvKp. Early identification of isolates carrying wild-type and mutated virulence genes can provide epidemiological data as well as appropriate management of the infection to reduce mortality. This test can provide an economical method for national surveillance studies, which will help to monitor the prevalence of hvKp.

The limitation of the present study includes the lack of diverse clones carrying aerobactin in the absence of *rmpA* and *rmpA2*. The *in vitro* and *in silico* models could not be validated using animals models to demonstrate the degree of virulence among the study isolates. Further studies involving patient outcomes and various genotypes of CR-hvKp are necessary to warrant the findings of this study. Also, of utmost importance is the genomic surveillance of CR-hvKp to determine the evolutionary changes in this pathotype.

## Conclusion

Increasing incidence of convergence of AMR and virulence is observed among Kp globally, which mainly occurs due to the uptake of a virulence plasmid by MDR clones or AMR plasmids by hvKp clones. Here, we report CR-hvKp in Indian subcontinent among MDR clones such as ST11, ST15, ST43, and ST231 that carry a virulence plasmid with non-functional RmpA2 but functional aerobactin. This highlights the significance of molecular/genomic identification of MDR-hvKp especially CR-hvKp and put in place better infection control measures to prevent the spread of this superbug. It is also imperative to determine the increasing incidence of this pathotype and monitor the clonal diversity from various geographical regions. The negative string test challenges phenotypic screening and rapid identification of CR-hvKp. Although classical RmpA2 is more stable than aerobactin, widespread mutations in *rmpA2* are accompanied with fitness cost to maintain non-functional RmpA2. On the other hand, high backbone stability and lower conformational fluctuations in the functional domain of aerobactin make it a better virulence factor that is retained among CR-hvKp. Therefore, aerobactin is a promising marker, as this superbug continues to evolve, reducing its fitness cost while coding for antimicrobial resistance and virulence.

**Table 3:** Virulence factors among classical and hypervirulent *Klebsiella pneumoniae* and their function.

Virulence factor	Function	Location in the genome	Classical *K. pneumoniae*	Hypervirulent *K. pneumoniae*
*allABCDRS*, *ybbW*, *ybbY*, *fdrA*, *fyuA*, *gcl*, *hyi*, KP1_1364, KP1_1371, *ylbE*, *ylbF*	Allantoinase cluster for Allantoin metabolism	Chromosome	X	√
*arc*	Arginine deiminase	Chromosome	X	√
*clbABCDEFGHIJKLMNOPQR*	Colibactin gene cluster	Chromosome	X	√
*iroBCDN*	Salmochelin	Virulence plasmid	X	√
*Irp1*, *irp2*	Yersiniabactin receptors	Chromosome	√	√
*iutA*, *iucABCD*	Aerobactin synthesis	Virulence plasmid	X	√
*kfuABC*	Iron uptake cluster	Chromosome	√/X	√
*glxK*, *glxR*	Glycerate pathway	Chromosome	X	√
*kvgA*, *kvgS*	Two-component regulator system regulating capsule synthesis	Chromosome	X	√
*mceABCDEGHIJ*	Microcin E492	Chromosome	X	√
*mrkABCDFHIJ*	Type 3 fimbriae	Chromosome	√	√
*rmpA*, *rmpA2*	Regulators of mucoid phenotype	Virulence plasmid	X	√
*ybtAEPQSTUX*	Yersiniabactin synthesis	Chromosome	√	√

X, absent; √, present; √/X, present/absent.

## Data Availability Statement

The datasets presented in this study has been submitted to NCBI and the accession numbers are provided in **Table 1**.

## Author Contributions

CS: conceptualization, *in-vitro* methods—WGS, analysis, manuscript writing. SB: *In silico* methods and analysis, manuscript writing. BL: Manuscript correction, supervision. SS: Isolate revival, PCR. KV: Bioinformatics methods—genome assembly. PM: Isolates, supervision. SR: Validation of methods and manuscript review. BV and AA: Conceptualization, project supervision, funding acquisition, manuscript review. All authors contributed to the article and approved the submitted version.

## Funding

This research was funded by the Indian Council of Medical Research (ICMR), Govt. of India, through the research grant IRIS-ID: 2019-0810.

## Conflict of Interest

The authors declare that the research was conducted in the absence of any commercial or financial relationships that could be construed as a potential conflict of interest.

## Publisher’s Note

All claims expressed in this article are solely those of the authors and do not necessarily represent those of their affiliated organizations, or those of the publisher, the editors and the reviewers. Any product that may be evaluated in this article, or claim that may be made by its manufacturer, is not guaranteed or endorsed by the publisher.
